# Associations between activity limitations and participation restrictions in people with late effects of polio

**DOI:** 10.1002/pmrj.70078

**Published:** 2026-02-24

**Authors:** Nilla Andersson, Katja Appelin, Jan E Lexell, Eva Månsson Lexell

**Affiliations:** ^1^ Department of Health Sciences Lund University Lund Sweden; ^2^ Department of Neurology, Rehabilitation Medicine, Memory disorders, and Geriatrics Skåne University Hospital Malmö Sweden

## Abstract

**Background:**

Late effects of polio (LEoP) are defined as new symptoms, for example, muscle weakness, fatigue, and pain, occurring many years after an acute polio infection. These impairments can lead to difficulties performing everyday activities (ie, activity limitations) that can reduce a person's ability to take part in society (ie, perceived participation restrictions). Yet, no study has explored how activity limitations are associated with participation restrictions in people with LEoP.

**Objective:**

To explore the association between activity limitations and participation restrictions among people with LEoP.

**Design:**

Cross‐sectional descriptive study.

**Setting:**

Outpatient rehabilitation clinic.

**Participants:**

One hundred two people with LEoP.

**Interventions:**

Not applicable.

**Main outcome measure(s):**

Canadian Occupational Performance Measure (COPM) assessing performance with everyday activities (COPM Performance) and satisfaction with performance in everyday activities (COPM Satisfaction), and Reintegration to Normal Living Index (RNL‐I) assessing perceived participation.

**Results:**

The mean scores of COPM Performance and COPM Satisfaction with performance were 5.3 and 5.0, respectively, and the mean score for RNL‐I was 71.6, indicating mild to moderate disability among the participants. The linear regressions, adjusted for gender and age, showed significant associations between COPM Performance and RNL‐I (*β* = 0.024, 95% confidence interval [CI] 0.001–0.039, *p* = .015) and between COPM Satisfaction with performance and RNL‐I (*β* = 0.024, 95% CI 0.003–0.046, *p* = .029). Significant associations were also found between the COPM subgroup self‐care and the RNL‐I subscale Daily functioning.

**Conclusions:**

The results show that people with LEoP perceive activity limitations as well as participation restrictions and that these concepts are only weakly associated. Therefore, during rehabilitation both activity limitations and participation restrictions need to be addressed in people with LEoP. This new knowledge may assist rehabilitation professionals when developing and implementing targeted interventions for people with LEoP.

## INTRODUCTION

Late effects of polio (LEoP), also referred to as postpolio syndrome,^1^, ^2^, ^3^ is one of the most common neuromuscular conditions worldwide. Globally, 10 million people have survived an acute polio infection during childhood and about 80% of those will later in life develop LEoP.^1^, ^2^ About 5000 people are living with LEoP in Sweden.^4^ New and progressive muscle weakness, muscular fatigue, general fatigue, and muscular and joint pain are common impairments among people with LEoP.^2^ These impairments are known to lead to activity limitations^5^, ^6^ and participation restrictions^7^, ^8^ and can also reduce the person's life satisfaction.^9^, ^10^

There is no cure for LEoP, but people who live with the condition can benefit from a period of interdisciplinary rehabilitation.^1^, ^2^ Comprehensive interdisciplinary rehabilitation, with the goal to decrease self‐reported disability through individually tailored interventions, has shown to be useful for people with LEoP. A qualitative study^11^ described how people with LEoP experienced improvements in managing their daily activities and viewed their future and self in a positive manner after having participated in an interdisciplinary rehabilitation. Participants with LEoP have also described that writing a rehabilitation plan based on the International Classification of Functioning, Disability and Health (ICF) is an important part of their rehabilitation process that assisted them not only to realize the scope of their problems but also what type of help they were able to receive.^11^, ^12^ Using an ICF‐based rehabilitation plan means that the consequences of LEoP—covering impairments, activity limitations, participation restrictions, and their interactions^13^—are in focus, where goals specifically address activity limitations and participation restrictions.

People with LEoP report a wide range of challenges performing various activities.^5^ Although many people with LEoP are independent in their personal daily activities (95%), dependence has been reported in more complex activities, such as cleaning (58%), shopping (53%) and transportation (69%).^6^ This is in line with another study suggesting that 51% need help with household work on a daily basis.^14^ Still, performing daily activities independently is not equivalent to performing them without problems. A newly published article states that people with LEoP report difficulties with self‐care (49%), productivity (29%), and leisure (22%).^15^ This confirms the results from other studies, showing that people with LEoP have significantly more problems in personal activities of daily living, such as bathing and tying shoelaces, than others^16^ and that >50% have different problems with walking.^17^ Participation restrictions have also been reported for this group. Although a majority of people reported sufficient participation in many activities, restrictions have been described in heavy household work, travel, and keeping a job.^7^ A qualitative study focusing on aging with LEoP also described work challenges, problems with household activities, less recreation, and fewer social activities.^18^

The association between certain areas of life has been described for people with LEoP. For example, low to moderate significant associations have been found between body functions and life satisfaction^10^ and between participation and life satisfaction.^9^ However, despite being central during rehabilitation for people with LEoP, there is very limited research regarding the association between activity limitations and participation restrictions. A better knowledge of the association between these ICF domains in people with LEoP may assist professionals to plan and implement interventions together with the person in need of rehabilitation.

Thus, the aim of this study is to explore the association between activity limitations and participation restrictions among people with LEoP. More specifically, we explored if participation restrictions are associated with activity limitations in terms of performance and satisfaction with performance and if limitations in self‐care, productivity, and leisure are related to participation restrictions such as daily functioning and perception of self.

## METHODS

### Study design

This is a cross‐sectional descriptive study based on retrospective data from a clinical register at a rehabilitation unit in a university hospital in southern Sweden. The study is part of a larger project focusing on people with LEoP who participated in individualized, comprehensive, interdisciplinary rehabilitation.^15^ The main goal of the comprehensive, interdisciplinary rehabilitation program was to decrease self‐reported disability through individually tailored interventions within an interdisciplinary team.^11^

### Ethics

The participants were informed about the data collection and signed an informed consent. The principles of the Helsinki Declaration were followed and performed in accordance with medical research involving human participants. The project was approved by the Swedish Ethical Review Authority (2020‐05313).

### Participants and recruitment

We obtained data from a clinical registry collected in a specialized postpolio outpatient rehabilitation clinic between 2004 and 2015. Eligible participants had a confirmed history of prior polio and clinically confirmed LEoP. Of all potential participants in the clinical register (*N* = 712), 336 did not need comprehensive rehabilitation and therefore received only single visits at the clinic, where they, for example, met with a physician or a physiotherapist and had not filled in the assessments. Thus, none of the assessments in focus for the current study were completed. The remaining 376 potential participants with LEoP received comprehensive team‐based rehabilitation, and assessments that covered different aspects of the ICF were completed.^19^ In the current study, we included participants from a previous study that evaluated activity limitations before and after rehabilitation.^15^ Of the 376 potential participants, 94 had not filled in the Canadian Occupational Performance Measure (COPM), 74 had incomplete COPM, and 106 only reported mean scores for COPM, and therefore comprehensive assessments were not performed. In total, 102 participants filled in the COPM on admission and at discharge, which comprised the final sample.

### Assessment of activity limitations

The COPM^20^ was used to assess activity limitations among the participants. Following a standardized procedure, the COPM is administered as a semistructured interview and assesses self‐perceived performance and satisfaction with performance in daily activities. The COPM covers three subgroups of daily activities: (1) self‐care, (2) productivity, and (3) leisure. In the first step of the COPM interview, the person identifies those daily activities that he or she finds difficult to perform. In the next step, the person assesses the importance of each identified activity on a 10‐point scale, ranging from 1 (not at all important) to 10 (extremely important). In the final step, the person prioritizes the most important activities. In each of these, the person rates performance of the activity (ie, COPM Performance) on a 10‐point scale, ranging from 1 (not able to do) to 10 (able to do extremely well) and satisfaction with performance of the activity (ie, COPM Satisfaction) on a 10‐point scale from 1 (not satisfied) to 10 (extremely satisfied). A mean score of the COPM Performance and the COPM Satisfaction is finally calculated for the prioritized activities.^20^ The psychometric properties of the COPM have been evaluated with satisfying results regarding validity, reliability, and responsiveness.^21^ In the current study, the COPM was administered by an occupational therapist together with each participant.

### Assessments of participation restrictions

The Reintegration to Normal Living Index (RNL‐I)^22^ was used to assess participation restrictions among the participants. RNL‐I is a generic tool that assesses perceived participation in daily life after illness or trauma. The RNL‐I has been described to assess participation according to the ICF^23^ and has been used in several populations^24^, ^25^, ^26^ including people with LEoP.^8^, ^27^ It consists of 11 items where each is rated with the following response options: 1 = does not describe my situation; 2 = somewhat describes my situation; 3 = partly describes my situation; and 4 = fully describes my situation. The RNL‐I can be summed into a total score, ranging from 1 to 100, where greater scores represent higher re‐integration and participation.^22^ It can also be divided into two sub‐scales: items 1–8 regarding Daily Functioning (DF) have a total score that range from 8 to 32, and items 9–11 regarding Perception of Self (PoS) have a total score that range from 3 to 12. The RNL‐I has been psychometrically evaluated regarding test–retest reliability among people with LEoP and has shown satisfying results.^27^, ^28^ The RNL‐I was self‐administered by the participants.

### Analyses

Descriptive statistics were used to calculate the participants' characteristics, mean scores (SD), and numbers (%).

To explore the associations between COPM and RNL‐I, baseline data were used and the analysis was performed in two steps. In the first step, two linear regression analyses were conducted with COPM Performance and COPM Satisfaction as the dependent variable and with the RNL‐I (total score) as the independent variable in both regression analyses. Assumptions for linear regressions were evaluated graphically (a linear relationship between the dependent and the independent variable, the variance of residuals was constant of all variables, and normal distribution of the residuals) and were fulfilled.^29^ All regression models were first made unadjusted and then controlled for age and gender.

In the second step, an explorative analysis emerged during the analyses process, that is, if any of the COPM subgroups (self‐care, productivity, and leisure) for COPM Performance and/or Satisfaction was specifically associated with any of the two subscales in RNL‐I (DF and PoS). Thereafter, linear regression analyses were made with all subgroups (self‐care, productivity, and leisure) for both COPM Performance and COPM Satisfaction as dependent variables, and where the two subscales of RNL‐I were independent variables, giving a total of 12 analyses.

Some of the RNL‐I data had one or two internal missing responses on an individual level; seven participants had missing responses on the RNL‐I (8.6%), and item mean substitution imputation^30^ was used to calculate a total sum score for those participants. The IBM SPSS Statistic 28 software (IBM Corporation, Armonk, NY, USA) was used to analyze the data and the level of statistical significance was set to *p* < .05.

## RESULTS

### Participants

In Table 1, data for the 102 participants are presented. Their mean age was 61 years (range 29–81 years), and >70% were women. The mean age at polio onset was 5.5 years and around 39 years (range 1–70 years) had passed until new symptoms had occurred. About two thirds of the participants lived with another person, half of the participants lived in a house, and about one third of the participants were still working. A majority (91%) used orthoses and 99% used some type of assistive device for walking.

**TABLE 1:** Characteristics of the included participants (*N* = 102).

Variables	Value
Age, y, mean (SD) min–max	60.8 (9.7) 29–81
Gender, men/women, *n* (%)	28/74 (27.5/72.5)
Age at acute polio, y, mean (SD) min–max	5.5 (4.6) 1–20
Y before onset of late effects of polio, mean (SD) min–max	38.8 (11.1) 1–70
Vocational situation, *n* (%)	
Work	30 (29.4)
Health insurance	34 (33.3)
Old‐age pension	35 (34.3)
Other	2 (2.0)
Living situation, *n* (%)	
Living with another person (partner, spouse, children)	67 (66.3)
Living alone	34 (33.7)
Accommodation, *n* (%)	
House	52 (51.0)
Apartment	49 (48.0)
Assistive devices, *n* (%)^a^	
Wheelchair (manual)	16 (15.7)
Wheelchair (powered)	19 (18.6)
Walker	32 (31.4)
Crutches	34 (33.3)
Orthopedic technical aids	93 (91.2)
Personal care	40 (39.0)
Household	25 (24.5)

^a^ Some participants used several assistive devices.

### Mean and total scores for COPM performance, COPM satisfaction, and RNL‐I participation

In Table 2, data on the mean and total scores for COPM Performance, COPM Satisfaction, and RNL‐I participation for the 102 participants are presented. The mean score of COPM Performance and COPM Satisfaction was 5.3 and 5.0, respectively. The mean total sum score for RNL‐I was 71.6, with 25.0 for the subscale DF and 10.0 for PoS.

**TABLE 2:** Activity and participation assessed with the Canadian Occupational Performance Measure (Performance and Satisfaction) and the Reintegration to Normal Living Index for the 102 participants with late effects of polio.

	Mean value (SD)
COPM (mean score, *n* = 102)	
COPM Performance	5.3 (1.8)
COPM Satisfaction	5.0 (2.2)
RNL‐I (total sum score, *n* = 81)	71.6 (21.7)
Daily Functioning	25.0 (5.2)
Perception of Self	10.0 (2.0)

*Note*: COPM Performance rated on a 10‐point scale (1 = not able to do − 10 = able to do extremely well) and Satisfaction (1 = not satisfied −10 = extremely satisfied). RNL‐I, possible sum score ranging between 1 and 100 points, subscale DF, total sum score range between 8 and 32 points; Subscale PoS, total sum score ranging between 3 and 12 points, where higher scores indicate greater reintegration and participation.

Abbreviations: COPM, Canadian Occupational Performance Measure; DF, Daily Functioning; PoS, Perception of Self; RNL‐I, Reintegration to Normal Living Index.

### Associations between COPM performance, COPM satisfaction, and RNL‐I

**FIGURE 1:**
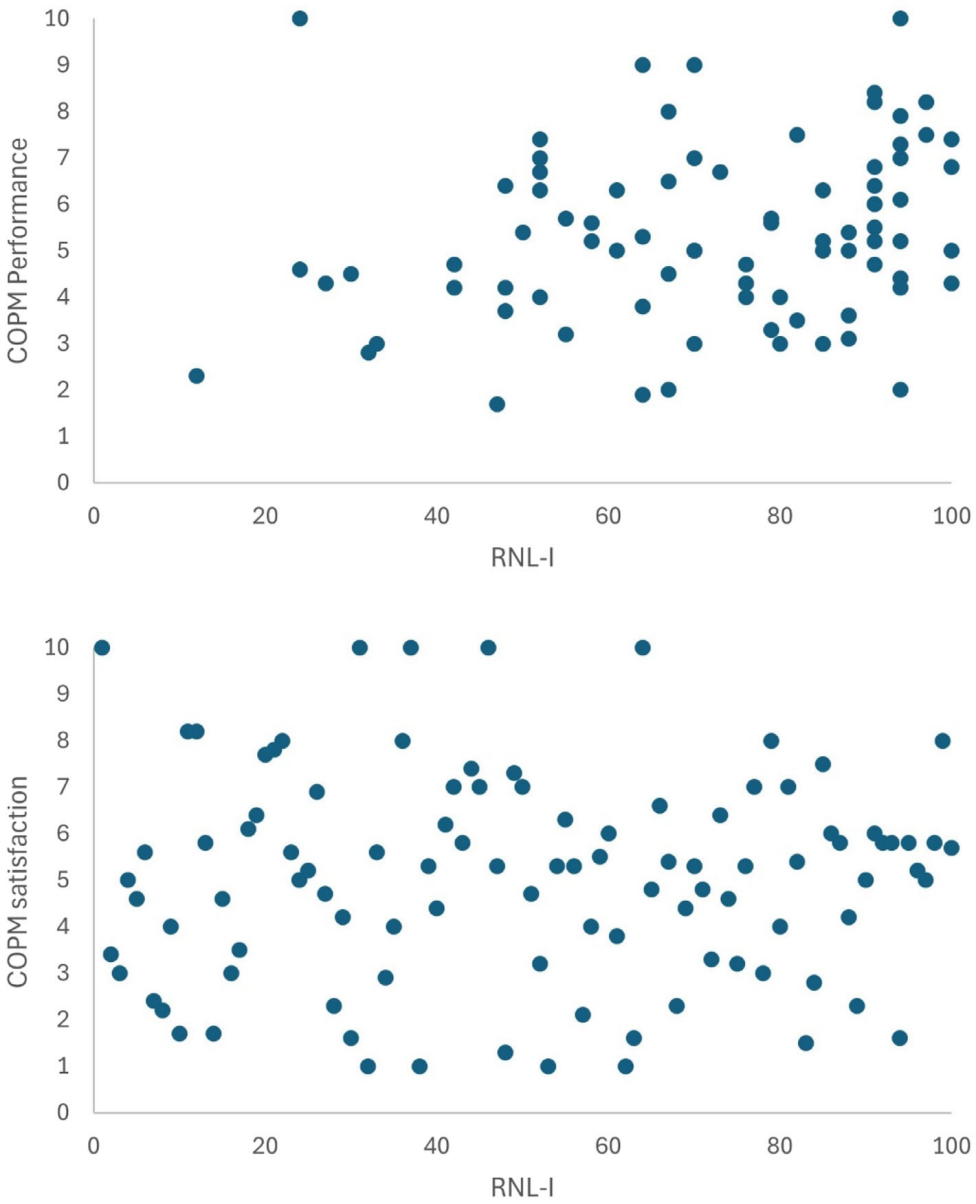
Scatterplot of the relationship between Canadian Occupational Performance Measure (COPM) Performance/Satisfaction and Reintegration to Normal Living Index (RNL‐I) for the participants (*N* = 102).

In Table 3, the result of the linear regression analyses between COPM Performance, COPM Satisfaction, and RNL‐I are presented. Both COPM Performance and Satisfaction were significantly associated with RNL‐I. When the analyses were controlled for gender and age, both COPM Performance and COPM Satisfaction were significantly associated with RNL‐I (*β* = 0.024, *p* = .015 and *β* = 0.024, *p* = .029, respectively). In Figure 1, the associations between COPM Performance and RNL‐I as well as between COPM Satisfaction and RNL‐I are graphically illustrated.

**TABLE 3:** Linear regression analyses of the association between COPM Performance, COPM Satisfaction, and RNL‐I participation for the 102 participants with late effects of polio.

Dependent variable	Independent variable	*ß* value (95% CI)	*p* value	*N*
COPM Performance	RNL‐I	0.020 (0.001–0.039)	.041	81
COPM Performance^a^	RNL‐I	0.024 (0.005–0.043)	.015	81
COPM Satisfaction	RNL‐I	0.018 (−0.004–0.040)	.109	81
COPM Satisfaction^a^	RNL‐I	0.024 (0.003–0.046)	.029	81

*Note*: COPM Performance, rated on a 10‐point scale (1 = not able to do − 10 = able to do extremely well)/Satisfaction (1 = not satisfied −10 = extremely satisfied); RNL‐I, total sum score range between 1 and 100 points, higher scores = greater reintegration.

Abbreviations: CI, confidence interval; COPM, Canadian Occupational Performance Measure; RNL‐I, Reintegration to Normal Living Index.

^a^ Analyses are adjusted for gender and age. A positive beta value indicates a positive association.

### Associations between the subgroups of COPM performance and COPM satisfaction and the subscales of RNL‐I participation

In Table 4, based on the explorative analyses the associations between the subgroups of COPM Performance and COPM Satisfaction and the subscales of RNL‐I participation are presented. One significant association was found, that between the subgroup self‐care of COPM Performance and the subscale DF of RNL‐I (*β* = 0.098, *p* = .026). No other significant associations were found for the other subgroups.

**TABLE 4:** Linear regression analyses exploring the associations of the subgroups of COPM and RNL‐I subscales for the 102 participants with late effects of polio.

Dependent variable	Independent variable	*ß* value, (95% CI)	*p* value
COPM performance	RNL‐I		
Personal care	DF (*n* = 75)	0.098 (0.012–0.184)	.026
	PoS (*n* = 75)	0.014 (−0.219–0.247)	.905
Productivity	DF (*n* = 58)	0.075 (−0.055–0.205)	.254
	PoS (*n* = 58)	0.165 (−0.174–0.504)	.333
Leisure	DF (*n* = 52)	0.069 (−0.078–0.215)	.351
	PoS (*n* = 52)	0.249 (−0.135–0.633)	.199
COPM satisfaction	RNL‐I		
Personal care	DF (*n* = 75)	0.073 (−0.028–0.175)	.154
	PoS (*n* = 75)	0.002 (−0.267–0.272)	.986
Productivity	DF (*n* = 57)	0.036 (−0.104–0.175)	.609
	PoS (*n* = 57)	0.156 (−0.200–0.512)	.384
Leisure	DF (*n* = 49)	0.100 (−0.064–0.263)	.226
	PoS (*n* = 49)	0.407 (−0.034–0.848)	.070

*Note*: COPM, Performance, rated on a 10‐point scale (1 = not able to do − 10 = able to do extremely well) and Satisfaction (1 = not satisfied −10 = extremely satisfied). Divided into the subgroups personal care, productivity, leisure. RNL‐I Index, subscale DF, total sum score range between 8 and 32 points; Subscale PoS, total sum score ranging between 3 and 12 points, at all higher scores = greater reintegration. A positive beta value indicates a positive association.

Abbreviations: CI, confidence interval; COPM, Canadian Occupational Performance Measure; DF, Daily Functioning; PoS, Perception of Self; RNL‐I, Reintegration to Normal Living Index.

## DISCUSSION

This study aimed to explore the association between activity limitations and participation restrictions among people with LEoP. The results show that people with LEoP perceive activity limitations as well as participation restrictions; the mean scores of COPM Performance and COPM Satisfaction were 5.3 and 5.0, respectively, and the mean score for RNL‐I was 71.6, indicating mild to moderate disability among the participants. It was also found that the RNL‐I was significantly, but weakly, associated with both the COPM Performance and COPM Satisfaction and that the subgroup self‐care of COPM Performance was significantly associated with the subscale DF of RNL‐I.

The main finding, that a greater perceived participation restriction (RNL‐I) was associated with a greater perceived activity limitation (COPM), is not surprising. Comparing our findings to empirical literature, the results are partially in agreement with those of McColl et al.^31^ The authors investigated the validity of the COPM, but not specifically in people with LEoP, and reported a significant but small association between COPM Satisfaction and RNL‐I for community living people who had occupational therapy services.^31^ The small association between the two assessments implies that the concepts—activity and participation—are related. This is also described in the theoretical literature that describes activity and participation as two different but related concepts. For example, ICF states that it can be difficult to distinguish between the two concepts and define them separately; activity is defined as the “the performance of a task or action by an individual” and participation as “engagement in a life situation.”^13^ Furthermore, the occupational therapy literature concludes that activity and participation are related and co‐constructive concepts that partly define one another.^32^, ^33^ Larsson‐Lund and Nyman^33^ also argue that engagement in activities supports participation and imply that activity is the means and participation is the outcome. Thus, the empirical findings from the current study support the existing literature on the relationship between activity and participation, as two concepts that to some extent are related yet not the same. Such knowledge is important to fully understand these aspects of the disability. As previously reported^5^ people with LEoP acknowledge problems in a wide range of activities, both in terms of how they perceived their performance and how satisfied they were with their performance. Still, when activity is addressed there is often a strong focus on performance^34^ and less on participation aspects. Our findings suggest that other focuses, such as taking part in activities, are also important to address. Clinically, this new knowledge implies that both activity and participation need to be addressed during the rehabilitation process among people with LEoP. This is in line with previous literature^19^ indicating that both concepts need to be addressed in an assessment phase of the rehabilitation process and included in the rehabilitation plan.

The explorative analyses showed a significant association between COPM Performance for the subgroup self‐care and the subscale DF in RNL‐I (*p* = .026). This is a new finding and at the same time also not surprising. The interview guide regarding the subgroup self‐care in COPM includes personal care activities as well as mobility and community mobility, activities that are also addressed in several of the items in RNL‐I subscale DF. Still, the association of the explorative analyses was weak, pointing out that each assessment tool has unique aspects that need to be addressed. Although no other associations were found in the explorative analyses, future studies need to address such associations in larger samples and validate these findings.

In the present study, COPM and RNL‐I were used to assess activity limitations and participation restrictions. These assessment tools—COPM and RNL‐I—have been used frequently in the past, for example, when measuring rehabilitation outcomes in different neurological populations,^35^, ^36^, ^37^ including people with LEoP.^15^, ^38^ The COPM has also been shown to be a well‐suited client‐centered assessment tool for identifying relevant activity limitations among people with different diagnoses on admission to rehabilitation.^39^, ^40^ Still, because the COPM is administered as a semistructured interview, the person may not identify problems with activities in all subgroups of the COPM,^5^ and problems may also change during the rehabilitation process.^15^ Furthermore, other aspects may influence COPM scores, such as acceptance of the disease and adaptation to the consequences in daily life that the disease may lead to.^15^ The COPM may therefore have some limitations when used as an outcome measure in research studies. Although the RNL‐I has been defined as a tool that assesses participation,^22^, ^28^ our results—showing a significant association (*β* = 0.098, *p* = .026) between COPM Performance and RNL‐I DF—suggest that the subscale DF mainly assesses activity limitations. For instance, item 1, “I am comfortable with how my self‐care needs are met,” covers aspects of activity.^22^ If, however, other assessment tools are used to evaluate activity and participation the results may turn out differently. It is therefore important to consider which concepts need to be addressed in research or in clinical follow‐ups and how these concepts are being operationalized by the chosen assessment tool.

As people with LEoP report a wide range of activity limitations with a diverse complexity,^5^ different aspects of activity and participation may be important to address during rehabilitation. That is, there are aspects of those concepts that are not covered by the definition of activity and participation in the ICF.^13^ For example, occupational balance is becoming an important aspect within rehabilitation and occupational therapy research, and for people with neurological disorders^41^, ^42^, ^43^ where occupational balance focuses on how satisfied the person is with the amount and variation of activities he/she has in daily life.^44^ Another aspect that can be of interest is if a person perceives activity gaps, that is, if there is a discrepancy between the activities that the person wants to perform and actually performs.^45^ Occupational gaps have been studied in people with acquired brain injury^45^ and stroke.^46^ However, none of these assessment tools has been used in people with LEoP but may be relevant due to their complex difficulties in everyday life.^5^, ^15^ Thus, evaluation of activity and participation is an unexplored area in relation to people with LEoP and further studies addressing different aspects of activity and participation are warranted.

### Strengths and limitations

A strength of this study is the number of included participants (*n* = 102) with clinically verified LEoP. Few participants with severe LEoP were included and therefore the results cannot be generalized to the entire LEoP adult population. The results are mainly representative for those with mild‐to‐moderate LEoP‐related disability. The study sample had a larger proportion of women (72.5%) compared to the general gender distribution usually reported for people with LEoP.^1^ There were also some missing data; 21 participants had not filled in the RNL‐I, which might affect the external validity of the results and should be taken into consideration when interpreting the results.

Another limitation is that there were few participants included in some of the explorative analyses of the study. For example, only 49 participants could be included in the analysis of COPM Satisfaction addressing leisure. As described in the discussion, this is a result of the administration of the instrument where the person identifies their self‐perceived difficulties in each activity, and leisure problems were not frequently reported in the data. Therefore, studies with larger samples capturing broader activity problems need to be pursued to confirm the results from the present study. Also, this exploratory study has a cross‐sectional design limiting the conclusion of the results. Future studies therefore need to address activity and participation from a longitudinal perspective and in a larger sample, as well as potential confounders to confirm this association.

Another methodological consideration is that when several statistical analyses are made, there is an increased risk for Type 1 error. Often corrections for multiple testing are used, for example Bonferroni correction. However, the Bonferroni correction is sensitive to Type 2 errors and could fail to recognize significant associations even if they exist.^47^ We chose not to use corrections for multiple testing and therefore advise caution when interpreting *p* values <.05 and rather see them as trends. Lastly, item mean substitution imputation was used for those that had one or two missing items on RNL‐I, which potentially can influence the results. However, we believe that the risk of it affecting the results is small as it involves a relatively small number of people (8.6%).

## CONCLUSION

The results show that people with LEoP perceive activity limitations as well as participation restrictions, and that these concepts are only weakly associated. Therefore, during comprehensive rehabilitation both activity limitations and participation restrictions among people with LEoP need to be addressed. This new knowledge may assist rehabilitation professionals when developing and implementing targeted interventions for people with LEoP.

## FUNDING INFORMATION

This study was funded by the Norrbacka‐Eugenia Foundation and the Promobilia Foundation.

## ETHICS STATEMENT

The study has been approved by the Swedish Ethical Review Authority (2020–05313). On admission to rehabilitation, each participant received information about the clinical database and gave their written informed consent to have their data included in the database. The study was performed in accordance with medical research involving human participants as described in the Declaration of Helsinki.

## DISCLOSURE

The authors have no competing interest to declare.

## Data Availability

The data used in the current study are not publicly available due to the sensitive information about the study participants. Furthermore, the participants did not provide consent for public data sharing (according to the Ethical approvals: Nos. 2020‐05313), but data can be available from the corresponding author upon reasonable request.
